# Temporal and Spatial Evolution of Eichmann Lake Wetland in Aksu River Basin and Its Response to Ecological Water Supply

**DOI:** 10.3390/ijerph20010351

**Published:** 2022-12-26

**Authors:** Yan Nie, Chen Yin, Pu Wang, Xingying He, Junjun Cao, Jing Yu

**Affiliations:** 1 Hubei Provincial Key Laboratory for Geographical Process Analysis and Simulation, Central China Normal University, Wuhan 430062, China; 2 Wuhan Institute of Landscape Architecture, Wuhan 430081, China; 3 Hubei Key Laboratory of Regional Development and Environmental Response, Hubei University, Wuhan 430062, China

**Keywords:** ecological water supply, water body index method, Aksu River Basin

## Abstract

Timely understanding and quantitative analysis of the changing trend in natural ecosystems in arid and semi-arid areas and their response to the ecological water supply process are of great significance for maintaining the health of oasis ecosystems. Taking the Eichmann Lake wetland of the Aksu River Basin in Xinjiang as the research area, the temporal-spatial distribution characteristics of the lake and the response of ecological water in recent years were studied based on remote sensing images and monitoring data. The results show that: (1) The water surface area of Eichmann Lake is shrinking, from 61.57 km^2^ in 1996 to 27.76 km^2^ in 2020. The changes in water surface area have experienced three stages: rapid decline, slow decline, and slow recovery. After the ecological water supply, the water surface area has obvious seasonal changes with hysteresis; (2) In areas with a low average water level, the ecological water supply has a significant impact on the groundwater level. The higher the water supply is, the higher the groundwater level will be. There is a significant lag effect between the change in the groundwater level and the response of the ecological water supply, which is 1–2 months; (3) The response characteristics of different natural vegetation to the ecological water supply were different in interannual, seasonal, and spatial contexts. The response of *Populus euphratica* to the ecological water supply is obvious, and its growth is the best within the range of 100–500 m from the water supply outlet. This research can provide the basis for the rational allocation of the Aksu River Basin’s water resources, and also act as a valuable reference for the restoration and reconstruction of surrounding vegetation in the Aksu River irrigation area.

## 1. Introduction

Inland lakes are an important part of water resources and determine the health and evolution process of the oasis ecosystem [1,2,3,4]. With the increase in water demand and the intensification of global climate change, the problem of water resources has become increasingly prominent [5,6,7,8,9,10]. Two-thirds of the global population faces severe water scarcity at least one month a year, and half a billion people face severe blue water scarcity all year round [11]. Whether in developed countries or in developing countries, the ecological environment problem has become a major issue restricting economic and social development [12]. The ecological water supply is considered to be one of the most effective measures for dealing with these environmental problems and is of great significance to the restoration of regional ecosystems and the sustainable social and economic development of arid and semi-arid regions [13].

In the 1970s, the 321-km river channel in the lower reaches of the Tarim River was cut off, the groundwater level in the lower reaches decreased significantly, the terminal lakes (Lop Nur and Taitema Lakes) dried up one after another, the vegetation state degraded over a large area, and was accompanied by desertification [14]. In 2000, an ecological water supply project was implemented in the lower reaches of the Tarim River. After a continuous ecological water supply, lakes and *Populus euphratica* in the lower reaches of the Tarim River gradually returned to the earlier ecosystem state. With the help of remote sensing and location monitoring data, many scholars have quantitatively studied the restoration of the natural ecosystem in the lower reaches of the Tarim River from the perspective of lake area, biodiversity, vegetation restoration, and groundwater depth [15,16,17,18,19,20]. Some scholars have also evaluated the benefits of ecological water supply projects with observations of vegetation structure, ecological value, and ecological water security [21,22,23]. However, study of the impact of the ecological water supply in the source stream of the Tarim River has been limited.

Because the basin is considered to be an appropriate scale for natural resource planning and management, comprehensive water resources dispatched at the basin level have become the main approach to ecological water delivery, enabling a fairer, more effective and sustainable use within the basin [24]. The Aksu River is one of the sources of the Tarim River, at the following coordinates: (40–41°35′ N, 78°47′ to 82°43′ E). It has a continental monsoon climate and a relative lack of water resources. In a drive for economic gain, some forests and grasslands have been converted into cultivated land, ecological water has been occupied, and important natural ecosystems have gradually degraded. As the largest freshwater lake in the basin, Eichmann Lake has shrunk rapidly in the past ten years, the connected lake areas have gradually evolved into many independent small lakes, and the groundwater level has decreased, which has greatly changed the ecological function of the basin. Since 2017, the main flood season and irrigation interval have been used to gradually carry out ecological emergency water replenishment to Eichmann Lake. The water level has since risen, the water quality has improved, the groundwater level has risen and the *Populus euphratica* forest has gradually recovered. Due to the short ecological water supply time, Eichmann Lake is still shrinking, and the natural forest grass vegetation still needs further restoration and improvement [25,26,27]. At the same time, there are few studies on the area changes, vegetation distribution patterns, and the water supply response by lakes in the Aksu River Basin. Understanding spatio-temporal changes in the lakes of the Aksu River Basin and evaluating improvements in environmental quality are important for the scientific guidance provided to the ecological water supply program in the basin, as well as for restoration projects [28,29,30,31,32]. Therefore, taking Eichmann Lake Wetland in the Aksu River Basin as the research area, combined with remote sensing data, groundwater level observation data, and ecological water supply data from 1996 to 2020, this paper analyzes the temporal and spatial evolution characteristics of Eichmann Lake wetland (mainly lakes and vegetation) and its response to the ecological water supply [33,34]. It will provide a reference for ecological protection and restoration, ecological water use management, and benefit future assessments of large-scale ecological water supply projects in the Aksu River Basin.

## 2. Materials and Methods

### 2.1. Data Sources

Landsat TM, GF-1 WFV image data covering the study area in 1996, 2005, 2010 and 2015–2020, and Gf-2 image data partially covering the lake area come from the geospatial data cloud (http://www.gscloud.cn/, accessed on 21 December 2022) of the China Satellite Application Center (http://www.cresda.com/CN/, accessed on 21 December 2022). ENVI 5.3 software was used to preprocess the original remote sensing image. Pretreatment include radiometric calibration, atmospheric correction, geometric correction, mosaic and clipping, image fusion to obtain the natural vegetation growth status in the study area with the help of a normalized vegetation index [28,29,30]. The data on groundwater depth dynamic monitoring and ecological water supply in the study area are provided by the Aksu administration of the Tarim River Basin.

### 2.2. Study Area

**Figure 1:**
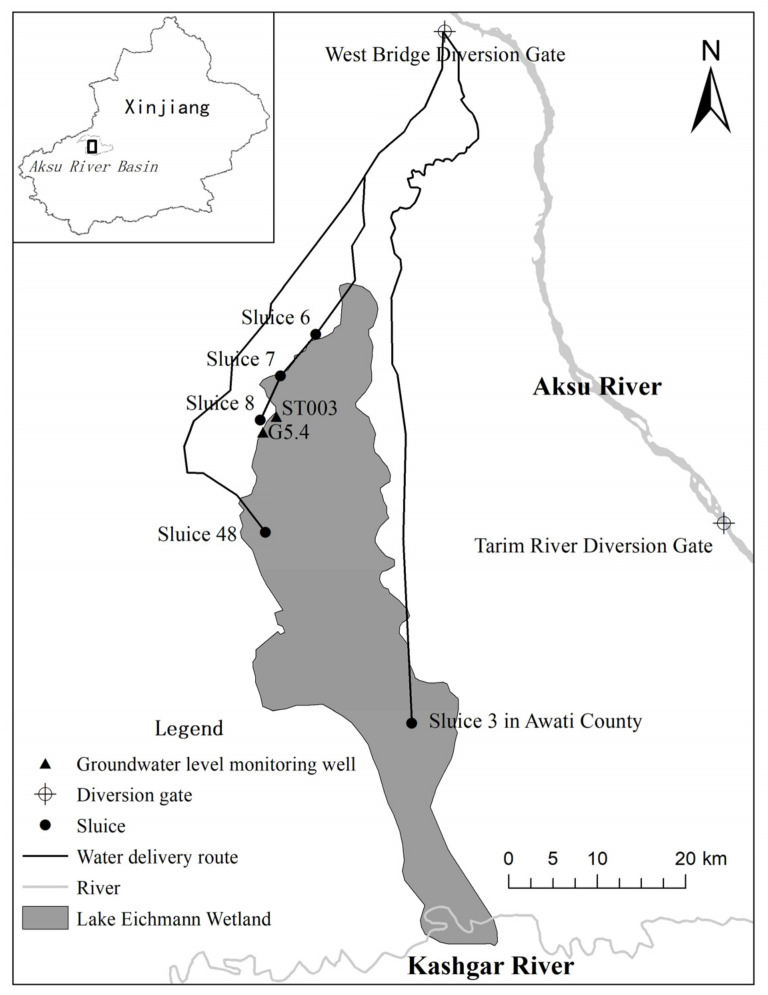
Map of Eichmann Lake wetland ecological sluice and groundwater level monitoring well.

Eichmann Lake is the earliest irrigation area for ecological water supply in the Aksu River Basin. The lake area is connected by eight small lakes, including Saylik Lake, Palace Lake and Moon Lake. It is the largest freshwater lake in the Aksu region of Xinjiang, one of the important habitats for migratory birds, and an important ecological component of the Aksu River Basin. Xinjiang Uygur Autonomous Region incorporated Aksu River into the ecological water supply area of the *Populus euphratica* forest key reserve in the Tarim River Basin for the first time in 2017, and the cumulative water delivery was 350.18 million m^3^ by 2020. From 2017 to 2020, a total of 228.5 million m^3^ of water was delivered to the Eichmann Lake area, mainly in August, and the water delivery time was about one month. There are three water delivery channels for the ecological water supply to Eichmann Lake: first, a water supply from the water distribution hub of West Bridge to Saylik Lake in the Eichmann Lake area through Shengli canal and Ayinko main canal, gate 6 and gate 7, and a water supply to Huanggong Lake in the Eichmann Lake area through gate 8; Second, replenish water to the Yituanhaizi wetland in Eichmann Lake area through the 48th sluice of Shengli canal; Third, the water from the water distribution hub of the west bridge passes through the old river to the first water diversion gate, and then passes through the yangwalik canal to the No. 3 gate of Awati county to replenish water to Eichmann Lake (Figure 1).

### 2.3. Methods

#### 2.3.1. Water Index Method

The water body index method [35] enhances the contrast between features by using ratios of remote sensing image bands (NDWI uses the second and fourth band pixel values, MNDWI uses the second and fifth band pixel values, EWI uses the first and fourth band pixel values). The commonly used water body indexes include the normalized difference water body index (NDWI), the improved normalized difference water body index (MNDWI) and the enhanced water body index (EWI). Among these, EWI has the best effect on extracting water in semi-arid areas [36,37]. Meanwhile, in 2013, Gaofen-1 satellite was launched and put into use. Through an experimental test, the Gaofen-1 water body index (GF1_WI) can more significantly highlight the effect of the water body through the contrast of absorption and reflection in the blue band and near the red band of the GF-1 WFV image. The calculation formula is:

(1) $$GF1\_WI=\frac{\left(B1-B4\right)}{\left(B1+B4\right)}$$

(2) GF1_WI>α

where B1 and B4 are the DN values of the first and fourth bands, respectively, and α is the threshold value. After many tests, the value of α was 0.

Therefore, this study selects the EWI to extract the lake water surface area in 1996, 2005 and 2010, and the GF1_ WI extracts the lake water surface area from 2015 to 2020. The accuracy of the extraction results is verified by 900 randomly selected samples. The results show that the verification accuracy is more than 90.6%.

#### 2.3.2. Time Series Change and Response Analysis

Interannual variation and seasonal variation are used to describe the temporal variation process of lakes. The interannual variation is analyzed by combining the annual maximum/minimum/average area curve of the lakes. The seasonal change is analyzed by the seasonal index method, in which the seasonal index is the relative number of monthly or quarterly changes in an index time series in a year calculated by the arithmetic average method.

In order to quantitatively describe the response of the lake area and vegetation index to the ecological water supply, the relationship between them was studied by trend analysis and spatial distance simulation. In addition, groundwater depth is the key factor for vegetation growth in arid areas [38]. In order to explore the impact of groundwater on the change in the index, the time lag correlation between the monthly vegetation index and the monthly groundwater curve is analyzed.

## 3. Results

### 3.1. Annual Variation Characteristics of Lake Water Surface and Its Response to Ecological Water Supply

**Figure 2:**
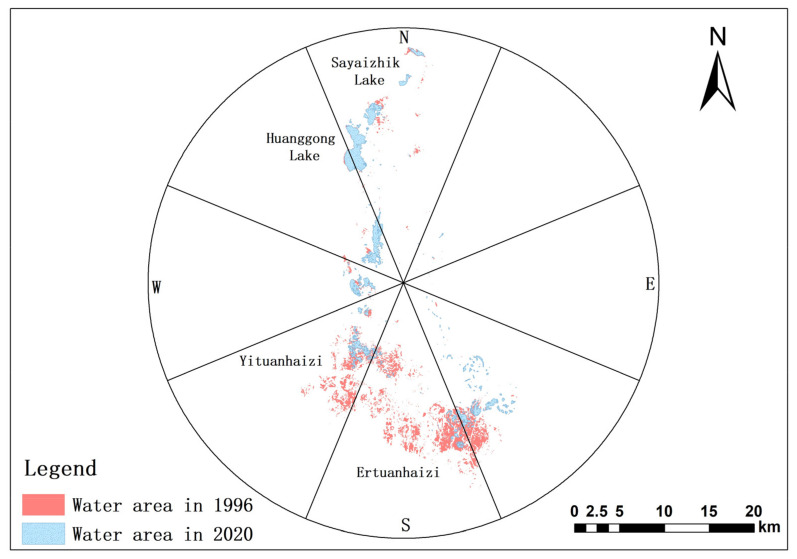
Schematic diagram of the spatial distribution of changes in the water surface area of Eichmann Lake.

The water surface area of the Eichmann Lake area in 1996, 2005, 2010 and 2015–2020 is extracted and summarized by the water body index. The results are shown in Table 1. On the whole, the water surface area of Eichmann Lake shows a significant decreasing trend. The water surface area was 61.57 km^2^ in 1996, and only 27.76 km^2^ in 2020, a total decrease of 33.81 km^2^ in 22 years, and the shrinkage rate of the lake is as high as 54.91%. According to the mathematical statistical analysis of the change in the land use structure of Eichmann Lake, the cultivated land area in the Eichmann Lake area increased from 66.86 km^2^ in 1996 to 116.88 km^2^ in 2020. The cultivated land area increased rapidly, the water demand for agricultural irrigation increased, the agricultural water occupied the ecological water, and the water entering the lake decreased. In addition, because residents illegally pump lake water for irrigation, the amount of lake water decreased, which is an important reason for the shrinking of the lake. Spatially (Figure 2), the water surface area of Eichmann Lake was mainly concentrated in the south of the lake in 1996. By 2020, due to the impact of ecological water replenishment, the water surface range of Sayaizhik Lake, Huanggong Lake, and Eichmann Lake in the north had not changed significantly, but the waters of Yituanhaizi, Ertuanhaizi, and Southeast in the west and south had shrunk significantly. At the same time, the area and speed of shrinkage in different directions are different. Except for the slight expansion in the due north and northwest directions, the lake water surface area shrank in the other seven directions, which is consistent with the decreasing trend in the total lake area.

To deeply analyze the change characteristics of the water surface area of Eichmann Lake in each period, the total change amplitude, annual change amplitude, and lake change intensity indicators are used to analyze the change in water surface area in each period. The results are shown in Table 2.

**Figure 3:**
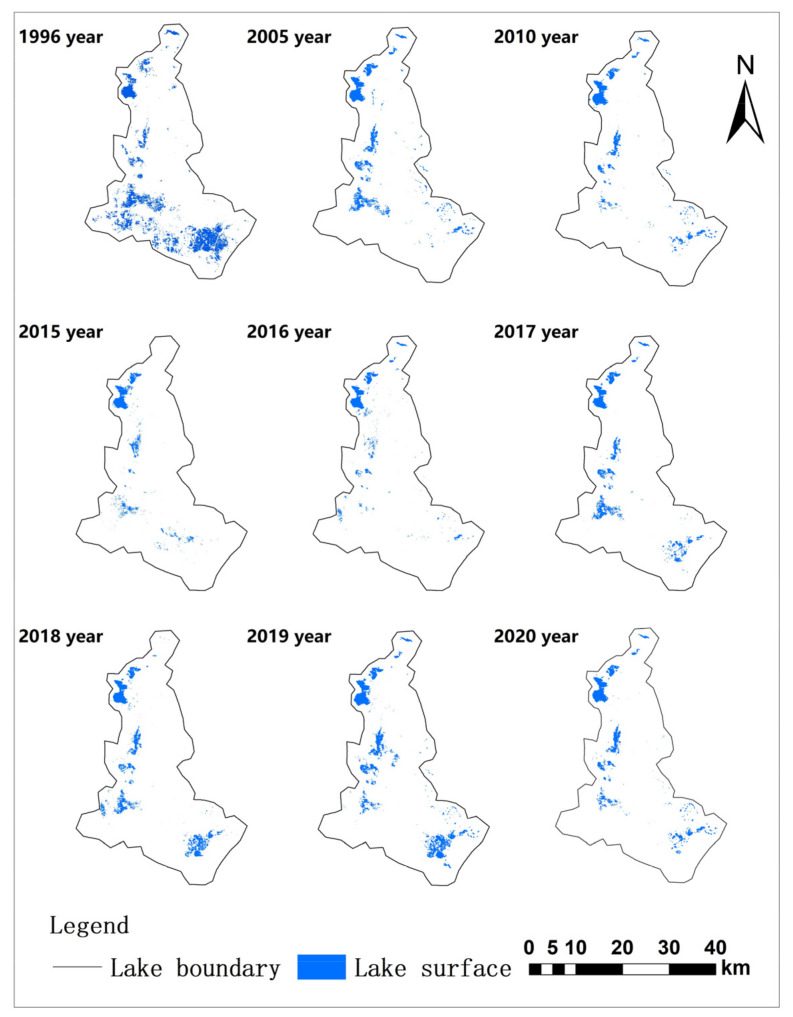
Schematic diagram of the time of changes in the water surface area of Eichmann Lake.

From the above results, it can be seen that the water surface area of Eichmann Lake decreased significantly from 1996 to 2020, showing a recovery trend in recent years, but it was still in a serious shrinking state. Generally, it can be divided into three stages: rapid decline, slow fluctuation decline, and slow recovery. (1) Rapid decline stage: from 1996 to 2005, the water surface area of the Eichmann Lake area decreased by 36.41 km^2^, the changing intensity of the lake was −6.57%, and the lake shrank rapidly. (2) Slow fluctuation decline stage: from 2005 to 2016, the water surface area decreased by 3.01 km^2^, and the changing intensity of lakes was −2.40%, which was significantly slower than that from 1996 to 2005. From 2010 to 2015, the water surface area of the lake area recovered, but the increase rate was very small. In 2016, the lake area continued to decline, with a decrease of 0.32 km^2^ compared with 2015. The changing intensity of the lake was −1.41%, and the reduction rate of the lake water surface area slowed down significantly. (3) Slow recovery stage: from 2015 to 2020, the water surface area of Eichmann Lake generally showed a fluctuating upward trend, and the lake began to recover slowly. Between them, 2017, 2018 and 2019 increased by 1.98, 3.36 and 6.47 km^2^, respectively, compared with the previous year, and the lake change intensity was 8.85%, 13.83% and 23.38%, respectively. The water surface area has gradually increased since 2017, and has reached 34.14 km^2^ in 2019. This is mainly due to the implementation of an ecological water supply policy for Eichmann Lake in 2017. After the water supply, the lake area increased significantly; the increase in the water surface is most obvious in Yituanhaizi, Ertuanhaizi (Figure 3), and the Southeast depression of the wetland. Ertuanhaizi formed the largest water surface in recent years in November 2018. The southeast depression is a seasonal stagnant lake, which started to form in 2017. It disappeared or froze in December of that year and reappeared in August or September of the next year. From 2017 to 2019, the water surface of the depression appeared earlier and was maintained longer, and the ecosystem restoration effect was obvious. (4) Secondary rapid decline stage: from 2019 to 2020, the water surface area of Eichmann Lake shrank slightly. This combined with the small reduction in the natural vegetation distribution area and NDVI value around Eichmann Lake in 2020, as can be seen from the China Meteorological data network (http://data.cma.cn/, accessed on 21 December 2022). The daily value data set of China’s surface climate data (V3.0) counted the annual data of precipitation in the Aksu River Basin from 2015 to 2020. The average precipitation monitored by the Aksu, Keping, and Alar observation stations close to the Eichmann Lake wetland was taken. The average annual precipitation from 2015 to 2020 was 107.33 mm, while the precipitation in 2020 was only 87.17 mm, the lowest value in the past six years. The drought in 2020 affected the growth of natural vegetation and the change in the lake water surface in the Eichmann Lake area. The change in climate factors led to a halt in the restoration of natural vegetation and lakes in the wetland in 2020, and the effect of the emergency ecological water supply was weaker than that in previous years. However, on the whole, the water surface area of the Eichmann Lake wetland showed a slowly increasing trend from 2015 to 2020.

### 3.2. Seasonal Variation Characteristics of Water Surface of Lakes and Its Response to Ecological Water Supply

**Figure 4:**
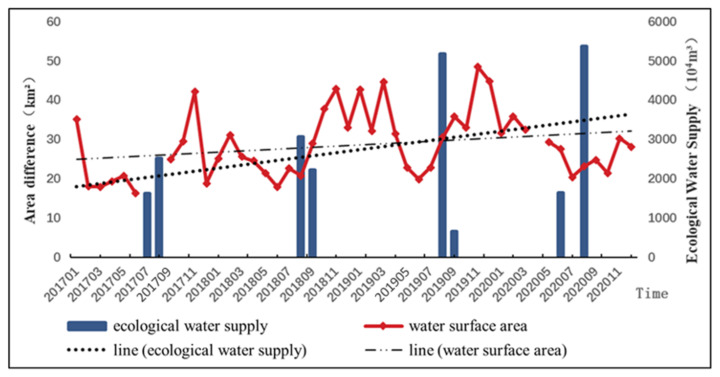
Plot of the monthly values of lake water surface area and ecological water supply quantities from 2017 to 2020.

The average value of the lake area in each month from 2017 to 2020, when the ecological water supply was implemented, was selected for further analysis (Figure 4). From the results, it can be seen that there are obvious seasonal changes in the water surface area of the lake after the implementation of the ecological water supply. From January to February, the water surface of the wetland shrank slowly with icing, increased slightly in March, and decreased continuously from April to June. The water surface gradually recovered from July to November, and the maximum water surface appeared in November. After November, the water surface began to shrink again. From July to September of each year from 2017 to 2020, it is the main month for the ecological water supply of Eichmann Lake. During this period, the water surface of Eichmann Lake was gradually expanding, but the maximum water surface area appeared in November. Mainly due to the large monthly evaporation of the Aksu River Basin from May to September, the water supplemented by the water supply to the lake was easily lost through the evapotranspiration of the lake surface, and the monthly evaporation of the basin decreased in November. The water demand from the natural vegetation and farmland production was also low, and the lake formed the largest water surface in the whole year.

In order to reveal the response relationship between lake surface area and ecological water supply, the monthly values of the lake area and ecological water supply from 2017 to 2020 are compared and analyzed respectively (Figure 2). It can be seen from the figure that there is an obvious lag between the month of ecological water supply and the increase in the lake water surface. The time of ecological water supply of the Eichmann Lake wetland is about August of each year from 2017 to 2020, and the water surface increased significantly in November. When we compare the difference between the monthly value of the water surface area and the annual average value of that year, it can be seen that the water supply effect in 2017 was clear, but the months in which the water surface area remained above the annual average value are only November and February of the following year. The water supply effect in 2018 and 2019 was clear and the effective maintenance time was relatively long, while the water supply effect in 2019 lasted until May of the following year. This shows that the ecological water supply has a positive impact on the restoration of the water surface of Eichmann Lake. The lake surface shrank again in 2020. The above analysis is related to the significant reduction in precipitation in 2020.

### 3.3. Dynamic Variation Characteristics of Groundwater Level and Its Response to Ecological Water Supply

**Figure 5:**
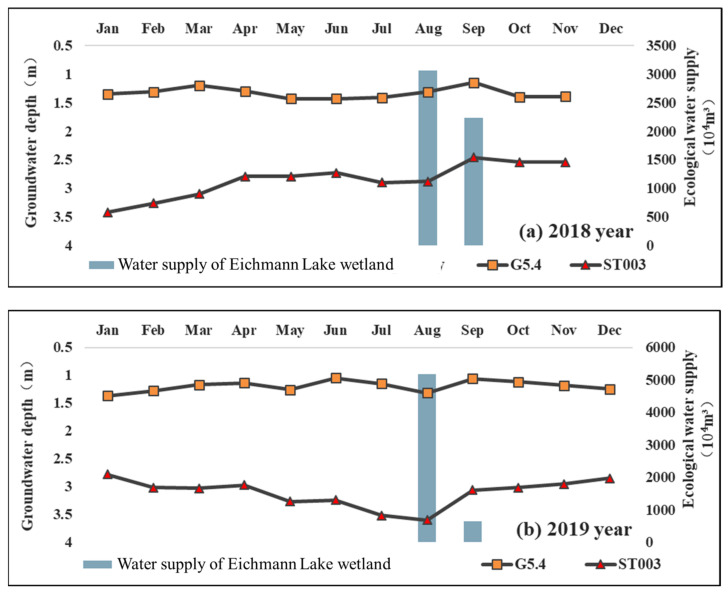
Observed changes in groundwater depth in monitoring wells under ecological water supply conditions in 2018 (**a**) and 2019 (**b**).

In order to dynamically monitor the change in the groundwater depth caused by the ecological water supply, two groundwater level monitoring wells, the G5.4 and ST003, were located in the water supply area of Eichmann Lake, located in the west of Eichmann Lake and close to the ecological water supply outlet. Due to the problem of automatic monitoring equipment, only the automatic monitoring data of the groundwater depth in 2018 and 2019 were selected for analysis (Figure 5). From the results, the groundwater depth of the G5.4 groundwater level monitoring well is relatively shallow, and the annual average burial depth in 2018 and 2019 is 1.335,1.196 m. The annual average groundwater depth of the ST003 groundwater level monitoring well is relatively deep, reaching 2.855 and 3.166 m in 2018 and 2019, respectively. The groundwater level monitoring well data show that the groundwater depth has an obvious response to ecological water transfer, especially in areas with low groundwater levels. In 2018, Eichmann Lake delivered 52.8977 million m^3^ of water continuously from August to September. The groundwater in the two groundwater level monitoring wells in Eichmann Lake rose significantly in September, with an average rise of 0.29 m; however, the groundwater level of G5.4 returned to the average level after September, and the groundwater level of ST003 rose until November (lack of data in December). In 2019, the groundwater depth of G5.4 and ST003 increased significantly with the ecological water transfer to Eichmann Lake in August. The variation characteristics of groundwater level of these two monitoring wells are consistent with those in 2018. Compared with August, the groundwater of G5.4 and ST003 increased by 0.26 and 0.54 m, respectively in September, with an average uplift of 0.4 m; the groundwater level of G5.4 decreased slowly after September. The area with deep groundwater level has a more obvious response to the ecological water supply than the area with shallow groundwater level.

In terms of seasonal variation, the groundwater level has obvious response characteristics to ecological water supply, and the response to ecological water supply is more obvious in areas with a low groundwater level, and the groundwater level maintains for a longer time after the water supply. The response to ecological water supply in areas with a high groundwater level is relatively slow, and it will soon return to the normal water level after the water supply; Affected by the difference in water level, the water level rise is more obvious in areas with a relatively low water level after the water supply. The rise in the groundwater level is related to water delivery. The water delivery to Eichmann Lake in 2019 increased by 5.3299 million m^3^ compared with that in 2018, and the rise in the groundwater level after water delivery increased by 0.11 m^3^. Many previous studies additionally believe that the ecological water supply is significantly related to groundwater uplift, and the closer it is to the water supply channel, the greater the uplift amplitude is, and the closer it is to the downstream [18,19,20]. At the same time, in the areas where the ecological water supply has been implemented, the lag time in the groundwater level response to the water supply may be shorter. The groundwater level of Eichmann Lake responds significantly in the second month of the water supply period, with a lag time of about 1 month. Previous studies [39] also believe that ecological water supply has a cumulative ecological effect and lag on groundwater level change, and the cumulative effect of the groundwater level change in the Tarim River is most significant after 80 days of water transfer.

### 3.4. Response Relationship between Natural Vegetation and Seasonal Variation of Groundwater Level

**Figure 6:**
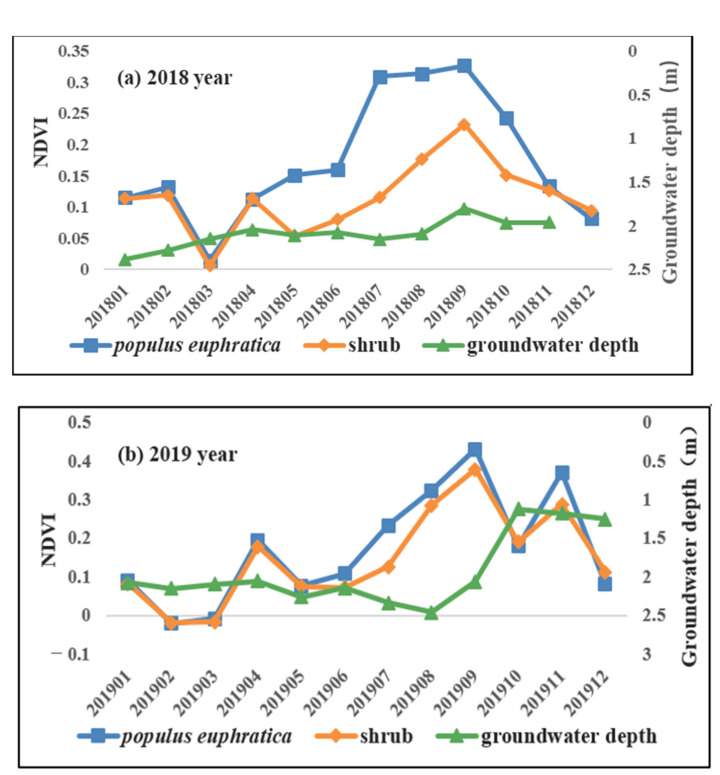
Comparison of groundwater level and natural vegetation NDVI in Eichmann Lake in 2018 (**a**) and 2019 (**b**).

Because there are few groundwater level monitoring wells in the study area and the time series data of groundwater depth monitoring are discontinuous, this study further analyzes the natural vegetation and groundwater level depth data of Eichmann Lake in 2018 and 2019. We extracted the monthly NDVI values of *Populus euphratica* forests and shrubs within 1000 m of the two groundwater level monitoring wells G5.4 and ST003 from 2018 to 2019, and analyzed the response relationship between natural vegetation and seasonal changes in the groundwater level (taking the average value of the two groundwater level monitoring wells G5.4 and ST003) in the Eichmann Lake area. Comparing the changes in natural vegetation NDVI and groundwater depth in the Eichmann Lake area (Figure 6), we found that the groundwater level in the Eichmann Lake area changed slightly in 2018, and that the rise in the groundwater level in the seasonal change occurred after the peak value of natural vegetation NDVI; The groundwater level in 2019 increased significantly in October and remained until December. The rise in the groundwater level in a seasonal variation also occurred after the peak of natural vegetation NDVI. However, the NDVI value of natural vegetation in the Eichmann Lake area rose again in November 2019, which may have been due to the high value of the groundwater level from October to December, resulting in a short rise in the NDVI of natural vegetation in November of the non-growth period in 2019. Studies have shown that the growth in natural vegetation in arid areas is affected by comprehensive factors such as groundwater, plant characteristics, and plant adaptation to the environment. Plants have a certain adaptation process to the rise or fall of the groundwater level. A long-term water shortage or sudden rise in the level in a short time may affect the growth environment of vegetation and disturb the growth changes of vegetation [7]. The results of this study also show that due to the limitation of water resources in arid areas and the seasonal differences in the demand for groundwater by the vegetation growth, the rise in the groundwater level often occurs in the later stage of the vegetation growth period. In the non-growth period, the large rise in the groundwater level will lead to a water stress effect on vegetation in a short time, which requires a certain adaptation time.

### 3.5. Response Relationship between Natural Vegetation and Ecological Water Supply

**Figure 7:**
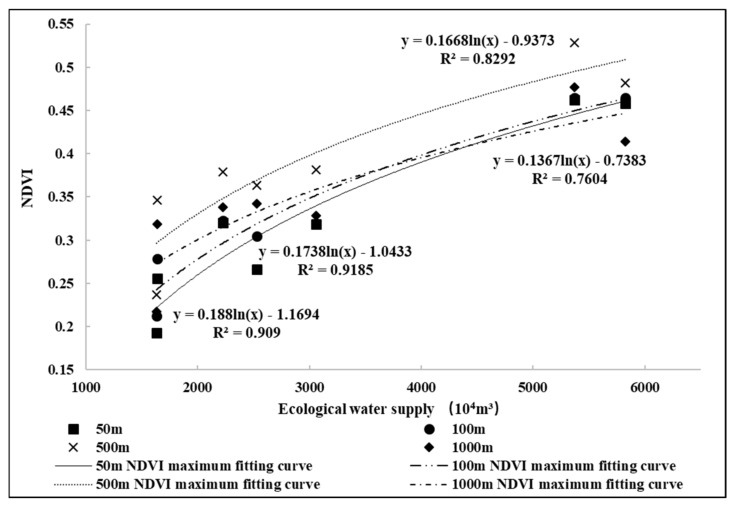
Fitting curve of relationship between ecological water supply and maximum NDVI of *Populus euphratica* forest in different distances.

By analyzing the relationship between the interannual change in the natural vegetation around Eichmann Lake and the response of the ecological water supply, we confirmed that from 2017 to 2020 the impact of the ecological water supply on the natural vegetation of the whole Eichmann Lake is not obvious, but that it has played an emergency role in the growth of the regional natural vegetation. In order to thoroughly analyze the impact scope of the ecological water supply and the temporal and spatial changes in its response relationship with natural vegetation, we extracted the NDVI values of the natural vegetation in different distances from the ecological water supply outlet in the Eichmann Lake area from 2017 to 2020, and fitted the maximum NDVI values of the natural vegetation after the annual water supply in different distances with the ecological water supply volume. Through many tests, different distances of 50, 100, 500 and 1000 m were selected for fitting (Figure 7). Figure 5 shows that the NDVI of *Populus euphratica* forest is the highest within 100~500 m of the water outlet, but that the fitting relationship between the maximum NDVI after water delivery and the ecological water supply is best within 100 m of the water outlet, and that the ecological water supply plays a more obvious role in the recovery of natural vegetation growth. There are differences between *Populus euphratica* forest and shrub in response to the ecological water supply. The goodness of fit between the maximum NDVI of *Populus euphratica* forest and the water supply volume is significantly better than that of shrub, which may be caused by the difference in root distribution between shrub and *Populus euphratica*, which directly absorbs soil water and groundwater by developing a large number of lateral roots, while the roots of shrub groups represented by Tamarisk and Haloxylon ammodendron grow downward. The water replenishment through the ecological water supply has little impact on deep water. Their different water absorption strategies lead to the more obvious response of *Populus euphratica* to the ecological water supply.

**Figure 8:**
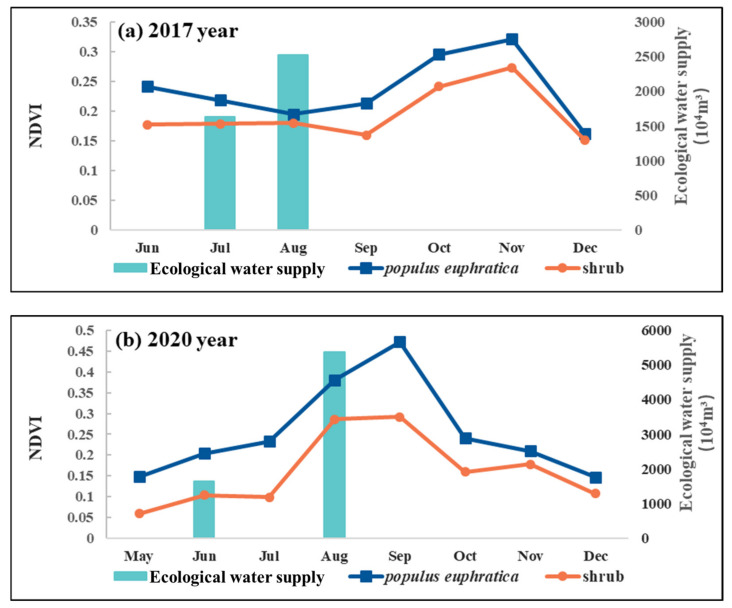
Changes in NDVI of the natural vegetation of Eichmann Lake before and after water delivery in 2017 (**a**) and 2020 (**b**).

In order to further explore the time variation law of the natural vegetation response to the ecological water supply, the NDVI of natural vegetation before and after the water supply in 2017 and 2020 were analyzed respectively. The maximum NDVI of *Populus euphratica* forest around Eichmann Lake has an obvious spatial distribution difference within 500 m of the water outlet. Therefore, the NDVI value of natural vegetation within 500 m of the water outlet was taken for analysis. Figure 8 shows that the ecological water supply was carried out for the Eichmann Lake wetland from July to August 2017; the NDVI value of the natural vegetation did not fluctuate significantly from July to August, and the NDVI value of shrubs increased slightly in August but decreased rapidly. The NDVI value of *Populus euphratica* forest and shrub did not increase significantly until October, and reached its maximum value in November. In June and August 2020, the ecological water supply was transported to Eichmann Lake. The ecological water supply volume in June was less than 20 million m^3^, and the NDVI value of natural vegetation fluctuated only slightly, which continued to decline from June to August. The water supply was carried out again in August, and the water supply volume reached 53.7303 million m^3^; the NDVI value of natural vegetation increased significantly in September. There were differences in water supply modes between 2017 and 2020. The total amount of water supply in 2017 was less, but it took 31 days to continuously convey water to Eichmann Lake from mid-July to mid-August, and the duration of the water supply effect was also relatively long. The total water delivery in 2020 increased by 28.5145 million m^3^ compared with that in 2017, of which the water delivery in June was small and lasted only 11 days; The water delivery volume in August was large and lasted for 18 days. The natural vegetation NDVI had an obvious response to the water delivery in the following month, but the duration was short. Combined with the results of fitting the analysis of the ecological water supply and NDVI maximum, the ecological water supply and water delivery time had an impact on the response between the natural vegetation and the ecological water supply. The greater the water delivery, the shorter the lag time of the natural vegetation response; the longer the time of continuous water supply, the longer the time of natural vegetation restoration after the water supply, and the more stable the vegetation restoration.

## 4. Discussion

In this study, because the time of the ecological water supply in the study area was short, there were few groundwater level monitoring wells, and the time series data on groundwater depth monitoring were discontinuous; the data with longer time and higher accuracy can be used to improve the analysis results in future studies.

At the same time, due to the scarcity of precipitation and large evaporation in arid areas, vegetation growth is extremely sensitive to water changes, and the impact of regional precipitation changes and soil moisture as well as other water factors on NDVI value changes cannot be ignored. In subsequent studies, the interannual and seasonal changes in regional precipitation should be included in the response analysis of the ecological water supply to improve the accuracy of the results.

In the fine classification of vegetation types, multi-source and multi temporal high-resolution data can also be introduced to improve the accuracy of research, especially to quantify the response lag time in ecological water transport [40].

## 5. Conclusions

(1) According to remote sensing data, the water surface area of Eichmann Lake has shrunk, from 61.57 km^2^ in 1996 to 27.76 km^2^ in 2020. The water surface gradually expanded from July, and the maximum water surface in the year appears in November. At the same time, the change in precipitation will damage the restoration of natural vegetation and lakes.

(2) The groundwater level clearly rises under the influence of ecological water supply, and has a lag effect. The time is about 1–2 months. The frequency and quantity of the ecological water supply affect the ecological water supply effect. At the same time, vegetation has a certain adaptation process to the rise in groundwater levels.

(3) The response characteristics of different natural vegetation to the ecological water supply is different in interannual, seasonal, and spatial contexts. The response of *Populus euphratica* forest to the ecological water supply is more obvious and the response lag time is shorter. The growth of *Populus euphratica* forest is best in the range of 100 ~ 500 m from the water supply outlet. The monthly change in the NDVI shows that the lag time of the ecological water supply to the growth of natural vegetation is about 1 month.

## Figures and Tables

**Table 1:** The water surface area of Eichmann Lake from 1996 to 2020.

**Year/a**	1996	2005	2010	2015	2016	2017	2018	2019	2020
**water surface area/km^2^**	61.57	25.16	22.15	22.65	22.33	24.31	27.67	34.14	27.76

**Table 2:** The change characteristics of the water surface area of Eichmann Lake from 1996 to 2020.

Interval/Yrs	Total Change/Km^2^	Annual Change/Km^2^	Relative Change/%
1996–2005	−36.41	−4.05	−6.57
2005–2010	−3.01	−0.60	−2.40
2010–2015	0.50	0.10	0.46
2015–2016	−0.32	−0.32	−1.41
2016–2017	1.98	1.98	8.85
2017–2018	3.36	3.36	13.83
2019–2019	6.47	6.47	23.38
2019–2020	−6.38	−6.38	−18.69

## Data Availability

Not applicable.
